# Fungal Rhinosinusitis: A Retrospective Microbiologic and Pathologic Review of 400 Patients at a Single University Medical Center

**DOI:** 10.1155/2012/684835

**Published:** 2012-02-08

**Authors:** Kathleen T. Montone, Virginia A. Livolsi, Michael D. Feldman, James Palmer, Alexander G. Chiu, Donald C. Lanza, David W. Kennedy, Laurie A. Loevner, Irving Nachamkin

**Affiliations:** ^1^Department of Pathology and Laboratory Medicine, Perelman School of Medicine at the University of Pennsylvania, 3400 Spruce Street, 6 Founders, Philadelphia, PA 19104, USA; ^2^Department of Otorhinolaryngology, Perelman School of Medicine at the University of Pennsylvania, Philadelphia, PA 19104, USA; ^3^Department of Otorhinolaryngology, The Sinus & Nasal Institute of Florida, St. Petersburg, FL 33716, USA; ^4^Department of Radiology, Perelman School of Medicine at the University of Pennsylvania, Philadelphia, PA 19104, USA

## Abstract

Fungal Rhinosinusitis (FRS) is a well known entity, but only in more recent times have the types of FRS been more fully defined. In this study, we evaluate the diagnosis of FRS in a single medical center. Cases were divided into 2 main categories, non-invasive and invasive. Non-invasive FRS included fungus ball (FB) and allergic fungal rhinosinusitis (AFRS). Invasive FRS included acute invasive fungal rhinosinusitis (AIFRS), chronic invasive fungal rhinosinusitis (CIFRS), and chronic invasive granulomatous fungal rhinosinusitis (CGFRS). Fungal culture data, if available was reviewed. 400 patients with FRS were identified. 87.25% were non-invasive (45% AFRS, 40% FB, and 2% combined AFRS and FB and 12.5% were invasive 11% AIFRS 1.2% CIFRS 0.5% CGFRS. One patient (0.25%) had combined FB/CGFRS. *Aspergillus sp.* or dematiaceous species were the most common fungi isolated in AFS while *Aspergillus sp.* was most common in FB and AIFRS. In our experience, most FRS is non-invasive. In our patient population, invasive FRS is rare with AIFRS representing >90% of cases. Culture data supports that a variety of fungal agents are responsible for FRS, but *Aspergillus sp.* appears to be one of the most common organisms in patients with FRS.

## 1. Introduction

Fungal rhinosinusitis (FRS) has been a known medical entity for several hundred years but only in more recent times the entity has been further defined. FRS comprises a variety of different disease processes which vary in presentation, histologic appearances, and clinical significance. Disease is most commonly classified as being non-invasive or invasive based on whether the fungi have invaded into the sinonasal submucosal tissue resulting in tissue necrosis and destruction [1–3]. Entities which are considered non-invasive FRS include fungal ball (FB (an entangled mass of fungus without significant surrounding sinonasal inflammatory reaction) and allergic fungal rhinosinusitis (AFRS) (a complex entity characterized by the presence of allergic mucin with histologic similarities to those reported in Allergic Bronchopulmonary Aspergillosis). Non-invasive FRS often requires surgical intervention and/or medical therapy. Invasive disease is characterized as either acute or chronic based on the length the time symptoms are present before presentation. Patients with acute invasive disease (AIFRS) are usually immunosuppressed and, by definition, present with symptoms of less than one-month duration. This entity is characterized by the presence of fungal forms invading into the sinonasal submucosal with frequent angioinvasion and rapid intervention is necessary. Patients with chronic invasive disease present with symptoms of greater than three month duration. Two forms of chronic invasive disease, chronic invasive FRS (CIFRS), and chronic granulomatous FRS (CGFRS), have been described and like AIFRS both are also serious, often requiring surgical and medical therapy. In the current study, we evaluate the histopathologic and microbiologic classification of a large population of FRS patients in a large quaternary care University Medical Center in the Northeastern United States.

## 2. Materials and Methods

The study took place at the Hospital of the University of Pennsylvania, a 725-bed academic quaternary care center. Following approval from the University of Pennsylvania Institutional Review Board, the Surgical Pathology archives were searched for patients with a diagnosis of FRS between the years 1991 and 2008. The surgical pathology reports and histologic slides were reviewed. The patients were classified as non-invasive (FB; AFRS and mixed FB/AFRS), invasive (AIFRS, CIFRS, and CGFRS), or mixed invasive/non-invasive based on known clinical and histologic criteria (Table 1). Results of fungal cultures, if available, were reviewed and correlated with the histopathologic findings.

## 3. Results

### 3.1. Patient Overview

Four hundred FRS patients were identified (Tables 2 and 3). 87.25% had non-invasive disease including 40.25% FB, 45.0% AFRS, and 2.0% combined FB and AFRS, and 12.5% had invasive disease, including 11.0% AIFRS, 1.0% CIFRS, and 0.5% CGFRS. One patient had combined non-invasive (FB) and invasive disease (CGFRS) (0.25%). Overall the mean patient age was 50 (range 18–90) with a male to female ratio of 1.2 : 1.

### 3.2. Noninvasive FRS

#### 3.2.1. Fungal Ball

**Figure 1:**
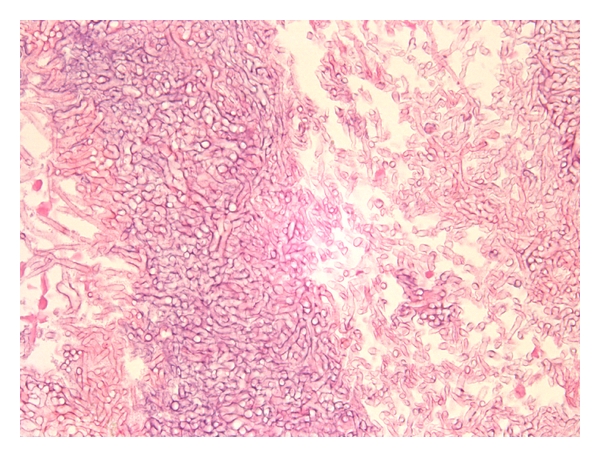
High power of fungus ball showing entangled masses of fungal organisms (original magnification ×200).

One hundred sixty-one patients were classified as having FB. Histologically, FB was characterized by the presence of entangled masses of fungal forms (Figure 1) [3, 4]. In many cases, fibrinopurulent material was seen surrounding the mass of fungi and the fungal organisms were identified embedded in acute inflammation and fibrinous exudate and only visible with histochemical stains (Grocott silver stain) for fungal organisms. In other cases, the fungal organisms were clearly visible on hematoxylin and eosin stains without significant surrounding inflammatory reaction. By definition, no tissue invasion or granulomatous reaction was identified [3, 4]. The average patient age was 55 (range 18–90), and there was a female to male ratio of 2 : 1. The disease was localized to one sinus in 97% of cases with no laterality predominance and was bilateral in 3% of cases. Seventy percent of cases involved the maxillary sinus and 27% involved the sphenoid/ethmoid sinuses. Fifteen patients had known sinus surgery 1 month to 14 years prior to diagnosis and an additional 14 subjects were immunosuppressed either by a malignancy or organ transplantation. Two patients showed recurrence of an FB, one at 6 months and the other at 7 years following the initial surgery.

One hundred seven patients had fungal culture results available for review (Table 4). Fifty-one percent had positive fungal cultures with *Aspergillus sp.* being the most common isolate. The remaining culture positive cases grew dematiaceous species as well as a variety of other fungi. Seven patients grew multiple fungal isolates in culture with 5 of 7 showing dematiaceous species in combination with other fungal organisms (Table 4).

#### 3.2.2. Allergic Fungal Rhinosinusitis (AFRS)

**Figure 2:**
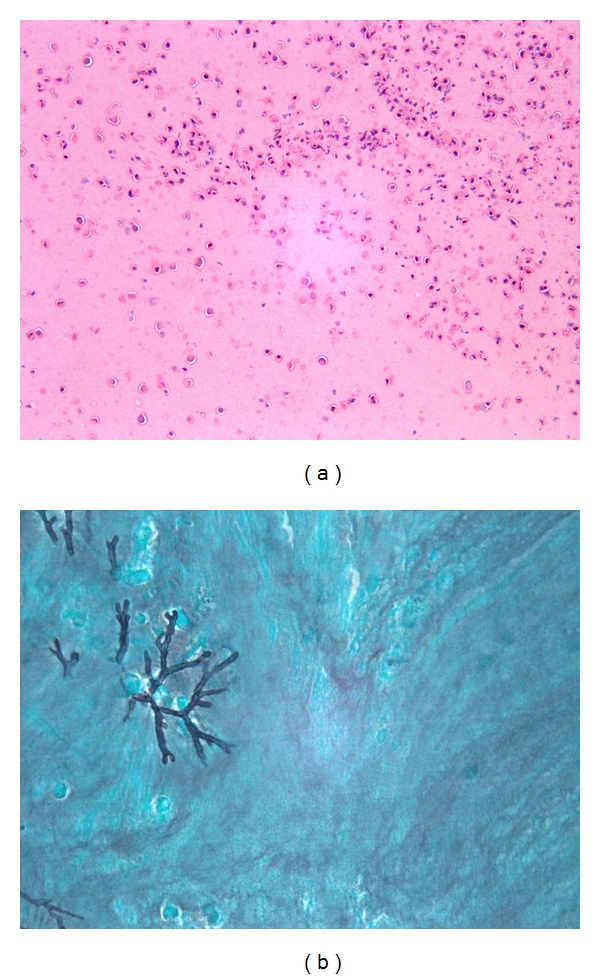
(a) Histologic appearances of EM with lamellated appearance of mucin admixed with eosinophils and eosinophilic debris (original magnification ×200). (b) Rare fungal forms present in EM in patient with AFRS which grew *Aspergillus sp.* in culture (original magnification ×200).

AFRS was diagnosed in 180 patients. The diagnosis of AFRS was established using pathologic criteria by either the presence of eosinophilic mucin (EM) containing fungal forms on histologic examination using a Grocott silver stain or the presence of EM without histologic evidence of fungi but with positive fungal cultures [5–7]. Histologically, EM was characterized by the presence of lamellated mucinous material with the presence of eosinophils, eosinophilic debris, and Charcot-Leyden crystals (Figure 2). In order to identify all potential cases of AFRS, all sinus contents specimens with histologically evident EM were reviewed. Three-hundred fifty-three patients with EM were identified, 134 of which had fungal forms by histology. Of the 219 patients without histologic fungus, 46 had positive fungal cultures for an overall total of 180 presumed AFRS patients. The remaining 173 patients with EM either had negative fungal cultures (100 patients) or unknown culture results (73 patients) and these patients were considered to have insufficient evidence for a pathologic diagnosis of AFRS although since more sensitive techniques for fungal detection such as PCR were not performed on this population, they may still actually have AFRS.

The average patient age was 45 (range 18–88) and there was a female-to-male ratio of 1.2 : 1. Thirty percent of patients presented up to 10 years prior to AFRS diagnosis with specimens showing chronic sinusitis with eosinophilia and/or the presence of EM without fungal organisms. Fungal culture results were available in 142 patients, 79% of which were positive (Table 5). Sixty-five percent of the culture-positive patients grew a single fungal isolate while 35% grew more than one fungal organism. The most common single fungal isolates were *Aspergillus sp.* (34%) with *A. fumigatus*, *A. flavus,* and *A. niger* most frequent and dematiaceous species (30%) with *Alternaria sp., Bipolaris sp.,* and *Curvularia sp*. isolated most often. Specimens from the remaining 36% of patients grew a variety of other fungi including *Paecilomyces sp.*, *Fusarium sp.*, *Scedosporium sp*., *C. albicans, and Penicillium sp*. Of the patients whose cultures revealed multiple fungal isolates, 34% grew a single dematiaceous species combined with a non*Aspergillus sp*., 23% grew a single dematiaceous species combined with *Aspergillus sp*. with or without another fungal species, 16% grew multiple *Aspergillus sp.* with or without a non-dematiaceous species, 14% grew multiple dematiaceous species with or without *Aspergillus sp*. and 13% grew neither *Aspergillus sp*. nor dematiaceous fungi. Forty-eight percent of patients with multiple isolates grew them at different points in time. One patient grew nine different fungal types in multiple specimens cultured over a three year time period.

There were noticeable differences between the fungi isolated in patients with EM containing fungal forms histologically and those patients with EM without histologically identifiable fungi but positive fungal cultures (Tables 6 and 7). In those AFRS cases with histologic fungi and positive fungal cultures, 84% had a single fungal isolate (35% *Aspergillus sp*., 36% dematiaceous species, and 13% other including *Fusarium sp. and Scedosporium sp.*); however fungal cultures in AFRS patients with positive cultures and EM without fungal forms showed a single isolate in only 63% of cases (24% *Aspergillus sp*, 10% dematiaceous species, 66% non-*Aspergillus*/non-dematiaceous species including *C. albicans, Penicillium sp,* and yeast not further specified).

#### 3.2.3. Mixed AFRS/FB

Eight patients had a mixture of FB and AFRS by histology. Six of these patients had evidence of entangled masses of fungus with extensive surrounding EM. Cultures were positive in four of these patients (*A. niger, A. flavus*, *Alternaria sp*. and *Paecilomyces* sp.) and were not performed in the other two patients. An additional, patient had an initial diagnosis of AFRS with negative fungal cultures with multiple subsequent surgeries showing EM but no fungal forms and cultures that were either negative or positive for *Alternaria sp, A. flavus* and *A. niger.* She then developed a FB 15 years subsequent to the initial surgery with fungal cultures positive for *A. flavus*. Similarly another patient had a diagnosis of AFRS with negative cultures and developed a FB culture positive for *A. niger* three years following the initial diagnosis of AFRS.

### 3.3. Invasive FRS

#### 3.3.1. Chronic Invasive FRS

**Figure 3:**
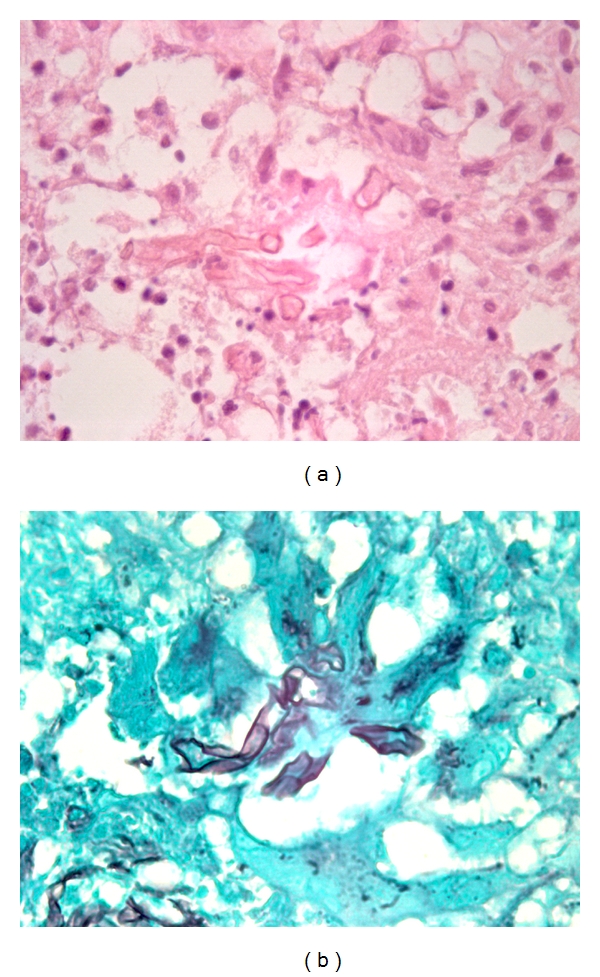
CGFS with granulomatous inflammation (a) and Grocott silver stain showing fungal organisms (b) (original magnification ×400 for (a) and ×400 for 3b).

There were six patients with chronic invasive disease, two CGFRS and four CIFRS. One of the CGFRS patients was a 50-year-old Sudanese female with an over 20-year history of CGFRS whose cultures grew *A. flavus*. The other CGFRS patient was a 66-year-old male with a long-standing history of non-insulin-dependent diabetes mellitus who presented with symptoms attributable to chronic rhinosinusitis. Cultures were not performed in this patient. Histologically, both specimens showed fibrosis, granulomatous inflammation, and chronic inflammation composed of lymphocytes and plasma cell without significant eosinophils (Figure 3).

**Figure 4:**
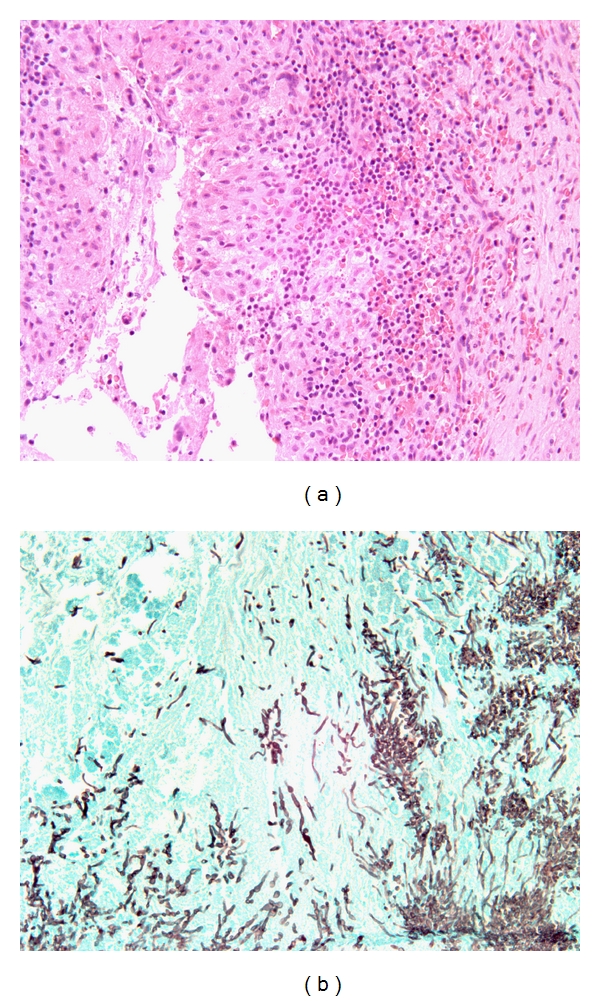
CIFS in showing chronic inflammation and fibrosis of the sinonasal mucosa (a) and fungal forms without angioinvasion invading into the sinonasal tissue on Grocott silver stain. (original magnification ×200).

The CIFRS patient population consisted of two males and two females. Two patients (ages 21 and 41) presented with long-standing nasal septal perforation of unknown etiology. Both of these patients showed mucosal ulceration with evidence of acute and chronic inflammation, fibrosis and submucosal invasive fungal forms without angioinvasion. Cultures grew *C. albicans* in one patient and were not performed in the other. The remaining two patients (ages 63 and 65) presented with a history of presumed chronic rhinosinusitis and no known risk factors for fungal sinusitis. Both of these patients showed chronic inflammation and associated mucosal ulceration with submucosal invasive fungal forms without angioinvasion (Figure 4). One patient grew *S. apiospermium* and cultures were not performed in the other.

#### 3.3.2. Mixed FB/CGFRS

One patient showed evidence of a FB with areas of underlying tissue invasion of fungal forms with the presence of granulomatous inflammation. This patient was a 72-year-old male who presented with long-standing diplopia, proptosis, and a left orbital “mass.” Histologically, the specimen consisted of a matted mass of fungal forms consistent with FB which was associated with the presence chronic inflammation with areas of submucosal fibrosis and focally granulomatous inflammation with fungal forms invading into the submucosal tissue without histologic evidence of angioinvasion. Cultures in this patient grew *A. fumigatus*.

#### 3.3.3. Acute Invasive FRS

**Figure 5:**
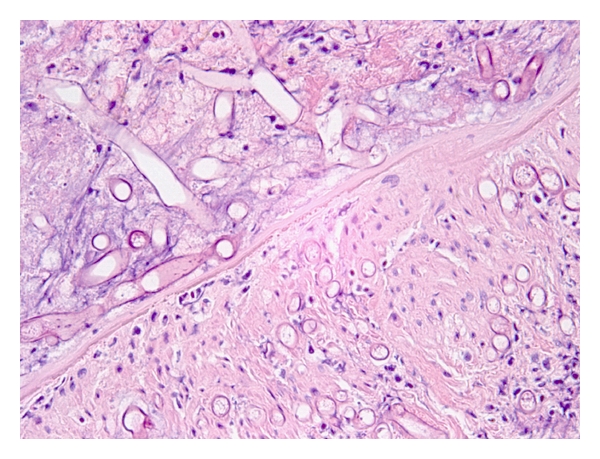
Fungal organisms invading into a large artery in a patient with AIFRS. Culture grew *Rhizopus sp*. (original magnification ×400).

Forty-four patients were classified as having AIFRS. Eighty-four percent had a hematologic disorder with or without bone marrow transplantation due most commonly to acute leukemia (32 patients), non-Hodgkin lymphoma (3 patients), and multiple myeloma (2 patients). The remaining seven patients had either treatment or conditions that led to immunosuppression including solid organ transplantation (2 liver and 1 kidney), systemic necrotizing vasculitis (1 patient), ulcerative colitis (1 patient) and non-insulin-dependent diabetes mellitus (2 patients). Patient ages ranged from 24 and 82 with a male-to-female ratio of 1.5 : 1. Fungal culture results were available in 27 patients with the most common cultured organism being *Aspergillus sp*. followed by *Rhizopus sp.* (Table 8). Other fungi isolated included one case each of *Alternaria sp*., *Paecilomyces sp*., and *Fusarium sp*. One-third of the patients had negative fungal cultures. Histologically all patients showed necrotic sinonasal mucosa with the presence of angioinvasive fungal forms (Figure 5).

## 4. Discussion

The diversity of FRS is highlighted by its many clinical and histopathologic presentations. Clinically, FRS can be acute (aggressive) and chronic (indolent) [1–3]. The pathologic spectrum encompasses a variety of different entities which are classified as either invasive or non-invasive and then into specific pathologic categories which are descriptive of clinical and histologic disease processes. In the current study, we present a cohort of 400 patients with FRS observed in a large quaternary care medical center that receives numerous referrals for surgical treatment of rhinosinusitis, in particular those with symptoms of chronic rhinosinusitis. As such, the majority of the patients that we observed were those with non-invasive disease with an almost equal distribution of AFRS and FB with incidences of 45% and 40%, respectively. In our experience, invasive FRS was rare with acute disease representing almost 90% of those with invasive disease. Chronic invasive disease was only rarely noted in our population and most likely reflects the referral population to our medical center. Relatively similar distributions have been seen in other studies performed in the United States. In a study from Houston, TX, Granville et al. reported non-invasive disease in >90% of patients with 72% AFRS, 23% FB, and 2.1% each of AIFRS and CGFRS [8]. In addition, Taxy observed non-invasive disease in over 80% of FRS patients in the Chicago area with an equal incidence of AFRS and FB and an 8% incidence of AIFRS [9]. Overall it appears, at least in these studies, that non-invasive FRS predominates in the United States population. However, studies performed in other countries such as India show significantly fewer FB cases and more CGFRS cases compared to these other studies. Das et al. observed non-invasive FRS in 60% of 284 patients (AFRS 56%; FB 4%) and invasive FRS in 36% of patients (AIFRS 17%, CIFRS 1%, and CGFRS 17%). [10]. Michael et al. observed a prevalence of 63% AFRS and 24% AIFRS similar to Das et al.. However, the incidence of CIFRS was higher (10%) and CGFRS significantly lower (<1%) than seen in Das et al.' study [11]. On the other hand, while Panda et al. observed a 60% incidence of non-invasive disease, they most commonly encountered FB in their series of 178 patients [12]. Challa et al. observed a much lower incidence of non-invasive FRS (25%) versus invasive disease (75%) with a 30% incidence of CGFRS [13]. This geographic diversity may be due to different climates and environmental factors, as well as different means of fungal exposure and variation in FRS terminology and definitions; however, the reasons for variations in FRS presentations are not understood.

Forty percent of the FRS patients observed in our study had FB. Panda et al. and Dufour et al. noted that FB was the most common form of FRS in their patients in India and France respectively, although several other studies as noted above have observed this form of FRS to be the least common [8–14]. The patient population we observed shared many features to those described in Dufour et al. with an increased incidence in elderly females, location in the maxillary sinus and the predominance of *A. fumigatus* on fungal cultures. However, our incidence of positive fungal culture was higher (51% versus 30%) than seen in their study and, in our patient population, we had only rare cases associated with *Scedosporium sp.*

AFRS was seen in 45% of patients in this study. We utilized basic pathologic criteria in categorizing AFRS patients [5–7]. AFRS was diagnosed as either the presence of EM containing fungal forms on histology (despite culture results) or the presence of EM without histologic fungi but positive fungal cultures. This latter group is probably the most problematic in the current study since it is difficult to ascertain without other clinical criteria that these patients should be truly considered as having AFRS. The diagnosis in this group was based on the presence of EM with positive fungal cultures; however, fungal cultures in this patient group commonly grew neither *Aspergillus sp.* nor dematiaceous fungi, two of the most common agents isolated in patients with AFRS, indicating that the fungi isolated may be unrelated to the EM and could be potential contaminants. It is possible that some of the patients with EM without fungi but positive cultures are not truly AFRS patients and that a positive fungal culture alone is not sufficient to make a diagnosis of AFRS without other clinical findings. The other problematic aspect of this study is that patients with EM but with no fungi on histology and negative fungal cultures were excluded. More sensitive fungal cultures, PCR, or other molecular techniques may have identified fungi in this patient group but, based on the current material available, a classification of these patients as having AFRS was not possible. Since this was a retrospective study, there was no way to control whether the EM material was preserved in a fashion to allow for more sensitive culturing techniques.

There has been recent controversy over the role of fungi in the development of chronic rhinosinusitis, an entity often treated with antibiotic therapy. In a study by Ponikau et al. using a sensitive fungal culturing technique, positive fungal cultures were seen in almost 100% of chronic sinusitis patients (although control subjects also had positive fungal cultures) [15]. While the finding of positive fungal cultures in most patients with chronic sinusitis is controversial in nature and not universally accepted, there is belief that chronic sinusitis may be related to inflammatory reaction to fungi [16–18]. Of note, almost 30% of the AFRS patients in this study initially presented with chronic sinusitis with eosinophils but no histologic evidence of fungus.

Fungi isolated in FRS, while showing geographic variation, are often similar in the particular forms of this disease. *Aspergillus sp.* particularly *A. fumigatus* and *A. flavus* appears to consistently be the most common agents isolated in patients with FB in a variety of different geographic locations including our current study [10–14]. Other fungal isolates including *Scedosporium sp., Fusarium sp.,* and dematiaceous fungi have also been rarely associated with this entity. *A. fumigatus, A. flavus*, and *Rhizopus sp.* are uniformly seen in patients with acute invasive disease worldwide [10–13]. Other rare fungi such as *Fusarium sp.* and dematiaceous species may also be isolated in AIFRS. Chronic invasive disease either CIFRS or CGFRS are also usually associated with *Aspergillus sp*, particularly *A. flavus* [10–13]. AFRS has the most variation in fungi isolated in different geographic locations. Most commonly, *Aspergillus sp*. and dematiaceous species are isolated but the species of fungi vary significantly from study to study. In studies performed in India as well as Saudi Arabia, *A. flavus* appears to be the most common fungal organism cultured in AFRS [10–13, 19]. In the United States, particularly in the South and Southwest, the majority of cases of AFRS grow dematiaceous fungi in culture. In Granville et al.'s study, almost 70% of AFRS cases grew dematiaceous fungi and their group showed no cases with *Aspergillus sp.* by culture [8]. Schubert and Goetz observed that >80% AFRS cases were associated with dematiaceous fungi with almost 70% due to *Bipolaris sp. *and only 9% due to *Aspergillus sp. *[20]*. *Manning and Holman reported dematiaceous fungi in almost 90% of their cases [21]. Alternatively, in the Chicago area, Taxy reported an equal incidence of *Alternaria sp.* and *Aspergillus sp.* in their patients with AFRS [9]. In the current study, both dematiaceous fungi and *Aspergillus sp*. were seen at relatively similar rates with *Alternaria sp., Bipolaris sp., Curvularia sp., A. fumigatus, A. flavus,* and *A. niger* being the most common single isolates in patients with histologically confirmed EM with fungal organisms. We also observed several patients with multiple different fungal isolates either at one time or multiple times during the disease process,. In 16% of patients with EM and histologic fungal forms, more than one fungal isolate was identified either at a single or at multiple time points, most commonly *Aspergillus sp*. or dematiaceous species with another fungus. This indicates that some patients may be predisposed to develop a reaction to more than one type of fungus including both dematiaceous and *Aspergillus sp.* This study did not control for the fact that the *Aspergillus sp*. may grow faster in culture than other organisms and that, even though *Aspergillus sp.* may have grown as an isolate in many AFRS patients, other types of fungi may also be present. Interestingly, in more recent studies, using in situ hybridization and immunohistochemistry, we have confirmed the presence of *Aspergillus sp.* in *Aspergillus sp*. culture confirmed allergic mucin containing fungal organisms (data not shown).

Mixed types of fungal sinusitis while uncommon can occur. In our study, 2% of patients showed a combination of FB and AFRS. In fact, two patients originally presented with AFRS and on subsequent surgery up to 9 years later developed a fungal ball. The other patients had classic EM with fungal organisms as well as an entangled mass of fungi consistent with FB. In addition, one patient in this study had combined FB and CGFS. About 4% of patients presented by Das et al. had combined FRS with either AFRS, FB, or AIFRS combined with CGFS [10]. Manning and Holman in a study from the Southwestern United States observed combined AFRS and CGFS in 1.5% of their patient population [21].

## 5. Conclusion

In summary, in this retrospective study, we present 400 patients with FRS. Non-invasive FRS was more frequently encountered in our patient population. Invasive disease, while less common in our group, was more commonly acute in nature. While a variety of fungi are isolated in patients with FRS, *Aspergillus sp*. still appears to be a common isolate in our patient population.

##  Disclosure

The authors have no financial and material support for this work. The authors have no financial interests or financial disclosures.

## Figures and Tables

**Table 1:** Classification of fungal rhinosinusitis.

	Histopathologic Criteria
Non-invasive FRS	
Fungus ball (FB)	An entangled mass on fungi with Minimal surrounding inflammatory reaction or surrounding fibrinous necrotic exudate containing fungal forms; no tissue invasion or granulomatous reaction is present
Allergic fungal rhinosinusitis (AFRS)	The presence of eosinophilic mucin (mucinous material admixed with eosinophils, acute inflammatory cells, eosinophilic debris, and Charcot-Leyden crystals; sparse fungi or positive fungal cultures; no tissue invasion present)
Mixed FB/AFRS	The presence of features of both AFRS and FB

Invasive FRS	
Acute (AIFRS)	Invasion of fungal forms into submucosal with frequent angioinvasion and necrosis in a patient with symptoms of less than one-month duration
Chronic (CIFRS)	Invasion of fungal forms into submucosal often with surrounding chronic inflammation and fibrosis in patient with long-standing symptoms (>3-month duration)
Chronic granulomatous (CGFRS)	Invasion of fungal forms into submucosal often with surrounding chronic inflammation, fibrosis, and granuloma production in patient with long-standing symptoms (>3-month duration)

Mixed Non-invasive/invasive FRS	A mixture of either of the invasive and non-invasive categories

**Table 2:** Classification of FRS in 400 patients.

Classification	Diagnosis	No. of patients	Total no. of patients	Percentage
Non-invasive			349	87.25%
	AFS	180		45.0%
	FB	161		40.25%
	Combined AFRS/FB	8		2.0%

Invasive			50	12.5%
	Acute	44		11.0%
	Chronic	4		1.0%
	Chronic granulomatous	2		0.5%

Non-invasive/invasive			1	
	Combined FB/chronic granulomatous	1		0.25%

**Table 3:** Clinical summary of FRS patients.

Diagnosis	Avg. age (range)	M : F	No.	No. with cultures (% positive)	Most common isolates (%)
FB	55 (18–90)	1 : 2	161	107 (51%)	*Aspergillus sp.* (66%)
AFRS	45 (18–88)	1.2 : 1	180	142 (89%)	Dematiaceous fungi (36%)
					*Aspergillus sp.* (35%)
AIFRS	54 (24–82)	1.5 : 1	44	27 (67%)	*Aspergillus sp.*
					*Rhizopus sp.*
CIFRS	48 (21–65)	1 : 1	4	2 (100%)	*C. albicans*
					*Scedosporium apiospermium*
CGFRS	58 (50–66)	1 : 1	2	1 (100%)	*A. flavus*

**Table 4:** Fungal Cultures in 161 Patients with FB.

Single fungal isolate (95.8%)	Culture result		Overall %
*Aspergillus sp*.			**65.8%**
	*A. fumigates*	44.8%	
	*A. flavus*	14.0%	
	*Aspergillus, *not* flavus or fumigatus *	7.0%	

Dematiaceous *sp*.			**9%**
	*Alternaria sp.*	5.0%	
	*Bipolaris sp.*	2.0%	
	*Trichophyton sp.*	2.0%	

Other			**21%**
	*Paecilomyces sp.*	5.0%	
	*Candida albicans*	2.0%	
	*Mucor sp.*	2.0%	
	Nonsporulating mold	2.0%	
	*Scedosporium sp.*	5.0%	
	*Penicillium sp.*	5.0%	

Multiple Fungal Isolates (4.2%)			
	*A. flavus/A. niger/Mucor* s*p. *	0.6%	
	*Alternaria/Penicillium*	1.2%	
	*Alternaria/Cladosporium/Penicillium/Trichoderma*	0.6%	
	*Paecilomyces/Fusarium/Curvularia/Alternaria*	0.6%	
	*A. flavus/A. NOS*	0.6%	
	*C. cifferinii/C. tropicalis*	0.6%	

**Table 5:** Fungal isolates in 127 AFRS patients.

			Overall %
Single Fungal Isolate			**65%**
*Aspergillus sp. *		34%	
*A. fumigatus *	41%		
* A. flavus *	25%		
* A. niger *	22%		
* A. terreus *	3%		
* A. NOS *	9%		
Dematiaceous *sp*.		30%	
*Alternaria sp. *	35%		
* Bipolaris sp. *	22%		
*Cladosporium sp. *	4%		
*Curvularia sp. *	35%		
*Scopulariopsis sp. *	4%		
*Paecilomyces sp. *		5%	
*Fusarium sp. *		6%	
*Scedosporium sp*.		5%	
*C. Albicans *		5%	
*Penicillium sp. *		5%	
*Yeast, NOS *		6%	
*Mucor sp. *		2%	
Mold, NOS		2%	
Multiple fungal isolates			**35%**
Dematiaceous species/non-* Aspergillus sp. *		34%	
*Aspergillus sp*./dematiaceous species ± other species		23%	
Multiple* Aspergillus sp.*/± other non-dematiaceous species		16%	
Multiple dematiaceous *sp*. ± other non-*Aspergillus sp. *		14%	
Non dematiaceous *sp*./non-*Aspergillus sp. *		13%	

**Table 6:** Fungal culture Results in 81 AFRS patients with EM and fungi seen microscopically.

			Overall %
Single isolate			**84%**
*Aspergillus sp. *		35%	
Dematiaceous species		36%	
Other single isolate		13%	
*Fusarium sp. *	6%		
*Scedosporium sp. *	3%		
*Mucor sp. *	1%		
Multiple isolates			**16%**
Dematiaceous species*/Aspergillus sp.*± other isolate		8%	
Dematiaceous species*/*non*-Aspergillus sp*.		7%	
*Aspergillus sp.*/non-dematiaceous species		1%	

**Table 7:** Fungal Culture Growth in 46 Patients With EM Without Fungal Organisms but with Positive Cultures.

			Overall %
Single isolate			**63%**
*Aspergillus sp*.		24%	
Dematiaceous species		10%	
Non-*Aspergillus*/non-dematiaceous fungi		66%	
*C. albicans *	17%		
Yeast, not *C. albicans *	14%		
*Penicillium sp*.	17%		
*Fusarium sp*.	3%		
*Paecilomyces sp*.	3%		
*Scedosporium apiospermium *	3%		
Mold not further specified	10%		
Multiple isolates			**37%**
Multiple dematiaceous species	12%		
Dematiaceous species*/Aspergillus sp.* ± other isolate	17%		
Dematiaceous species*/*non*-Aspergillus sp*.	41%		
*Aspergillus sp*./non-Dematiaceous species	12%		
Non-dematiaceous species/non-*Aspergillus sp*.	17%		

**Table 8:** Positive culture results in 18 AIFS patients.

		Overall %
*Aspergillus sp.*		49%
*A. Fumigatus *	56%	
*A. flavus *	33%	
Aspergillus, NOS	11%	
*Rhizopus sp.*		33%
*Fusarium sp.*		6%
*Paecilomyces sp.*		6%
*Alternaria sp. *		6%
